# Quality of Maternal Health Care: A Call for Papers for a Maternal Health Task Force–PLoS Collection

**DOI:** 10.1371/journal.pmed.1001134

**Published:** 2011-11-29

**Authors:** Samantha R. Lattof, Mary Nell Wegner, Ana Langer, and the PLoS Medicine Editors

**Affiliations:** 1 Maternal Health Task Force, Women and Health Initiative, Department of Global Health and Population, Harvard School of Public Health, Boston, Massachusetts, United States of America; 2 Independent Consultant, Maternal Health Task Force, Boston, Massachusetts, United States of America

## Abstract

The PLoS Medicine Editors and partners from the Maternal Health Task Force at the Harvard School of Public Health announce their partnership and a call for papers on “quality of maternal health care.”

## Progress to Build On

With four years remaining before the world is held accountable to the Millennium Development Goals (MDGs) and with numerous recent global pledges, it is imperative that energy and resources for maternal health remain focused to create the change needed, especially in the most vulnerable settings where maternal and newborn mortality and morbidity remain far too high. There are key advances on which to capitalize. Important progress has been made in reducing maternal mortality in specific locations [1]–[4]—the overall number of women dying from pregnancy and childbirth each year declined from 526,300 in 1980 to about 273,500 in 2011 [2], although the large declines in central Europe and south Asia are overshadowed by negligible progress in all regions of sub-Saharan Africa. Cost-effective and evidence-based interventions to prevent and successfully manage all major causes of maternal morbidity and mortality are available, including good nutrition, access to contraception, skilled attendance at delivery, and emergency obstetric care [5], although coverage of these known interventions is regrettably uneven [5],[6]. The field now has high-level political commitment and a more reliable scientific understanding of what it will take to prevent deaths among women and children worldwide. These forces have created an unprecedented opportunity for accelerating progress to achieve maternal health goals.

Vexing our road to success, however, is the continued lack of access to knowledge on maternal health in many settings, which is a major limitation to all of human development. As several large publishers withdraw from making their publications free to those in developing countries [7],[8], the Maternal Health Task Force (MHTF) (http://maternalhealthtaskforce.org/) is concerned that the chasm between those who have access to information on maternal health and those who do not will widen. The MHTF's overarching goal is access: more people should have greater access to more comprehensive maternal health information and knowledge. In order to achieve our goal of greater access, the MHTF is collaborating with PLoS to create a freely available, open access collection of published research and commentary on maternal health care: the MHTF–PLoS Collection on Maternal Health.

During the MHTF's first phase at EngenderHealth, a leading international reproductive health organization (www.engenderhealth.org), the Task Force brought together hundreds of global and country organizations to debate and reach consensus on critical issues related to maternal health using creative and participatory mechanisms, providing a platform for developing countries to lead and participate in technical exchanges and agenda-setting processes. The Task Force also mentored a cohort of young leaders, and identified and supported innovation in maternal health around the world.

To address the large set of continued needs identified through extensive consultations with its partners, the MHTF has recently moved to the Women and Health Initiative at Harvard School of Public Health (http://www.hsph.harvard.edu/women-and-health-initiative), where it will continue its work with a three-year, US$12 million project funded by the Bill & Melinda Gates Foundation that aims to improve global maternal health policies and programs by expanding access to critical knowledge; providing a neutral space for scientific debate and consensus building; strengthening the capacity of developing country professionals; and helping improve the content and quality of maternal health care. This second phase is based on a thorough analysis of the critical factors needed to accelerate progress in maternal health.

While there are many approaches to take, the MHTF–PLoS Collection in 2011–12 will focus on quality of maternal health care, as it is clear that such a focus is now a global imperative [9]. The quality of maternal health care is highly variable and often extremely poor, even in locations where there is a commitment to improving maternal health through increased access to skilled care, for example [10]. The coverage and reach of maternal health services have increased dramatically in some high-burden countries, but the content and quality of those services (both institutional and at the community level) have not improved at the same pace. For example, Stephen Lim and colleagues recently noted that the Janani Suraksha conditional cash transfer program in India, which provides incentives for women to give birth in a health facility, increased the number of institutional deliveries but did not improve the content and quality of care that the women receive [11]. In order to reduce maternal morbidity and mortality, it is becoming clear that policies and programs need to improve the quality on the supply side of the service delivery equation.

## A Call for Papers

For this MHTF–PLoS Collection on Maternal Health we welcome primary research (both quantitative and qualitative) and incisive commentary related to quality of maternal health services broadly, and more specifically in the following areas that have been identified by the global maternal health community as critical needs: (1) evaluation of behavioral interventions to facilitate the development and introduction of evidence-based practices by frontline health workers, and (2) evaluation of the content and quality of maternal health care through the use of sensitive, feasible, and simple indicators to monitor and evaluate health services' and systems' performance, especially as they relate to maternal health care. Ideally, research articles should not be merely descriptions of activities but should include evaluation of the impact of initiatives after their implementation. Commentary articles on quality of maternal health care, targeted to the *PLoS Medicine* Essay or Policy Forum sections, must be novel and well-argued. We consider maternal health an integral part of women's reproductive health and, therefore, papers linking care during pregnancy, delivery, and postpartum with other aspects of reproductive health will be relevant to the Collection.

All papers should be submitted to *PLoS Medicine*, with a note that they are intended for the Maternal Health Collection. Authors from developing countries for whom the publishing fee for research articles presents a barrier are encouraged to apply to the MHTF for financial support or to apply for the normal PLoS fee waiver. An initial decision will be made about papers' potential suitability for either *PLoS Medicine* or another PLoS journal. The authors will be informed of this decision, and papers will then be peer-reviewed according to the specific journal's policies. PLoS will retain all control over editorial decisions, and peer review will be conducted in accordance with the usual processes of the appropriate PLoS journal. Editorial staff has no knowledge of an author's ability to pay publication fees, so ability or not to pay cannot affect decisions. Once a paper is accepted for publication in a PLoS journal it will be forwarded to the selection panel for the Collection. This panel, which will be composed of PLoS and MHTF staff, will decide on articles' suitability for inclusion in the Collection.

We invite submissions now on the theme of quality of maternal health care. Articles will stand the best chance of inclusion in the 2011–12 Collection if they are submitted by 1 April 2012. Further information on this process is available at the PLoS Collections page (www.ploscollections.org/maternalhealth).

PLoS and the MHTF look forward to collaborating on this new initiative and hope it will encourage researchers to submit to PLoS their best research and commentary to help accelerate progress on the quality of maternal health care.

## Abbreviations

MHTF: Maternal Health Task Force
